# Supplementary material for: An extracellular matrix-related prognostic and predictive indicator for early-stage non-small cell lung cancer
Source: Nat Commun. 2017 Nov 23;8:1734. doi: 10.1038/s41467-017-01430-6 (PMC5700969; doi:10.1038/s41467-017-01430-6)
Supplement: Supplementary file 1 — Supplementary Information [file 41467_2017_1430_MOESM1_ESM.pdf]

	Gene Symbol	logFC	P.Value	adj.P.Val
**1**	*COL11A1*	2.663813678	5.57E-48	1.79E-46
**2**	*CLDN18*	-2.655746145	2.58E-51	1.19E-49
**3**	*ADH1B*	-2.581876586	1.63E-46	4.80E-45
**4**	*MMP12*	2.574903494	1.18E-45	3.22E-44
**5**	*WIF1*	-2.506725855	7.70E-40	1.25E-38
**6**	*SFTPC*	-2.495682052	1.37E-34	1.44E-33
**7**	*FABP4*	-2.445039233	4.71E-64	7.53E-62
**8**	*TMEM100*	-2.434622234	2.44E-59	2.39E-57
**9**	*SCGB1A1*	-2.383422791	1.86E-22	8.18E-22
**10**	*SPP1*	2.334613484	5.69E-62	6.97E-60
**11**	*GKN2*	-2.29543194	7.08E-57	5.57E-55
**12**	*AQP4*	-2.26575711	8.51E-36	9.83E-35
**13**	*MMP1*	2.236508033	4.42E-31	3.63E-30
**14**	*CPB2*	-2.221414391	9.71E-46	2.67E-44
**15**	*GREM1*	2.204186809	3.00E-43	6.65E-42
**16**	*CYP4B1*	-2.169222561	1.20E-36	1.48E-35
**17**	*AGER*	-2.136906649	8.83E-88	4.45E-84
**18**	*GJB2*	2.087157453	1.76E-42	3.62E-41
**19**	*SFTPA2*	-2.051344116	7.03E-24	3.40E-23
**20**	*CLIC5*	-2.037431569	3.03E-63	4.42E-61
**21**	*ADRB1*	-2.030868236	3.25E-52	1.63E-50
**22**	*TOP2A*	2.014367341	4.67E-60	4.75E-58
**23**	*MAMDC2*	-2.013077713	4.22E-49	1.51E-47
**24**	*HS6ST2*	1.995895019	1.32E-45	3.59E-44
**25**	*SFTPD*	-1.952742143	7.74E-32	6.73E-31
**26**	*ABCA8*	-1.952485506	1.91E-54	1.17E-52
**27**	*COL10A1*	1.947206526	3.66E-60	3.85E-58
**28**	*FAM107A*	-1.94635245	1.24E-75	1.39E-72
**29**	*MCEMP1*	-1.935165034	7.33E-63	9.59E-61
**30**	*TNNC1*	-1.930838473	1.15E-68	3.75E-66
**31**	*FCN3*	-1.920689657	7.14E-64	1.11E-61
**32**	*AKR1B10*	1.913926947	9.01E-22	3.79E-21
**33**	*MT1M*	-1.907263845	2.46E-54	1.49E-52
**34**	*GPM6A*	-1.902624456	2.71E-81	6.84E-78
**35**	*KRT6A*	1.866798256	9.34E-17	2.87E-16
**36**	*PEBP4*	-1.862824127	7.19E-44	1.69E-42
**37**	*SDPR*	-1.861487998	2.86E-58	2.53E-56
**38**	*SCN7A*	-1.844848022	6.65E-46	1.85E-44
**39**	*INMT*	-1.839407425	5.76E-57	4.61E-55
**40**	*CXCL13*	1.79184175	2.13E-28	1.42E-27
**41**	*HBB*	-1.770917395	2.14E-50	8.89E-49
**42**	*ANLN*	1.770778898	1.98E-50	8.24E-49
**43**	*TCF21*	-1.765967795	5.32E-63	7.25E-61
**44**	*CD36*	-1.747831139	2.10E-56	1.54E-54
**45**	*IGF2BP3*	1.742751736	3.98E-33	3.72E-32
**46**	*SCGB3A2*	-1.739967765	4.84E-17	1.52E-16
**47**	*LRRK2*	-1.739188517	5.16E-39	7.76E-38
**48**	*CHRDL1*	-1.738466927	8.99E-45	2.27E-43
**49**	*CTHRC1*	1.733147754	1.19E-58	1.10E-56
**50**	*SPINK1*	1.732941354	1.10E-17	3.60E-17
**51**	*HHIP*	-1.721958364	4.86E-50	1.94E-48
**52**	*PPBP*	-1.720413923	9.10E-43	1.93E-41
**53**	*COL6A6*	-1.717423112	6.29E-52	3.07E-50
**54**	*C2orf40*	-1.710910503	1.15E-41	2.21E-40
**55**	*GPX2*	1.70520818	1.59E-21	6.60E-21
**56**	*CEACAM5*	1.701245182	1.24E-17	4.02E-17
**57**	*FOSB*	-1.699225633	9.52E-36	1.10E-34

**58**	*S100A2*	1.698578459	5.57E-24	2.71E-23
**59**	*PGC*	-1.68449976	2.24E-25	1.20E-24
**60**	*C7*	-1.67456594	4.74E-36	5.55E-35
**61**	*TFAP2A*	1.664894295	7.51E-44	1.75E-42
**62**	*FMO2*	-1.662338116	3.43E-57	2.81E-55
**63**	*OGN*	-1.66062051	7.98E-40	1.29E-38
**64**	*STXBP6*	-1.657343316	9.05E-56	6.25E-54
**65**	*VEPH1*	-1.657255304	3.57E-49	1.28E-47
**66**	*ADAMTS8*	-1.656059102	3.61E-64	6.28E-62
**67**	*FAM150B*	-1.655821883	3.88E-63	5.42E-61
**68**	*PTPRB*	-1.653827903	1.05E-63	1.57E-61
**69**	*CDCA7*	1.650737609	1.12E-53	6.42E-52
**70**	*DLGAP5*	1.646162212	2.66E-48	8.79E-47
**71**	*PDK4*	-1.640954973	2.31E-40	3.90E-39
**72**	*TMPRSS4*	1.634397285	6.10E-46	1.70E-44
**73**	*FHL1*	-1.622980044	1.58E-56	1.19E-54
**74**	*ADIRF*	-1.619882459	1.48E-55	9.88E-54
**75**	*BTNL9*	-1.609885457	1.78E-77	3.00E-74
**76**	*IL6*	-1.60063726	5.39E-37	6.84E-36
**77**	*SOX7*	-1.599491119	6.29E-50	2.45E-48
**78**	*SOSTDC1*	-1.595241831	1.56E-33	1.50E-32
**79**	*SUSD2*	-1.577682473	1.85E-31	1.57E-30
**80**	*ANKRD29*	-1.573911876	6.79E-47	2.05E-45
**81**	*PSAT1*	1.570113621	1.94E-48	6.50E-47
**82**	*CXCL2*	-1.564517289	1.85E-36	2.25E-35
**83**	*NCKAP5*	-1.561720003	4.64E-70	1.73E-67
**84**	*ASPM*	1.560963466	3.35E-46	9.47E-45
**85**	*ABI3BP*	-1.558353841	3.87E-47	1.19E-45
**86**	*LPL*	-1.555105676	1.21E-44	3.04E-43
**87**	*PLA2G1B*	-1.550192724	9.57E-37	1.19E-35
**88**	*SLC2A1*	1.542417609	8.54E-42	1.65E-40
**89**	*CDC20*	1.542063794	3.39E-49	1.22E-47
**90**	*GINS1*	1.536462223	1.09E-54	6.89E-53
**91**	*RRM2*	1.531842112	7.07E-48	2.27E-46
**92**	*TPX2*	1.531554798	7.34E-47	2.20E-45
**93**	*EDNRB*	-1.530877603	3.94E-72	1.97E-69
**94**	*ACADL*	-1.517576379	1.61E-57	1.34E-55
**95**	*LAMP3*	-1.517435283	4.40E-44	1.06E-42
**96**	*NEK2*	1.5157416	1.49E-47	4.72E-46
**97**	*MELK*	1.510749552	1.37E-49	5.06E-48
**98**	*CA4*	-1.504747964	1.68E-93	1.70E-89
**99**	*MARCO*	-1.504515354	2.99E-51	1.36E-49
**100**	*AGTR1*	-1.503517517	9.94E-54	5.72E-52
**101**	*S100A12*	-1.502949761	1.52E-40	2.64E-39
**102**	*PCOLCE2*	-1.502567684	8.48E-43	1.80E-41
**103**	*LEPR*	-1.501624592	6.39E-47	1.94E-45

Supplementary Table 1. 103 differentially expressed genes in 925 primary NSCLC tumors compared to 193 normal lung tissues from merged microarray data

Division	*Category*	*Gene Symbol*	*Full Name*	*logFC*	*P.Value*	*Adj.P.Value*
Core matrisome	Collagens	COL11A1	collagen, type XI, alpha 1	2.663814	5.57E-48	1.79E-46
Core matrisome	Collagens	COL10A1	collagen, type X, alpha 1	1.947207	3.66E-60	3.85E-58
Core matrisome	Collagens	COL6A6	collagen, type VI, alpha 6	-1.71742	6.29E-52	3.07E-50
Core matrisome	ECM Glycoproteins	SPP1	secreted phosphoprotein 1	2.334613	5.69E-62	6.97E-60
Core matrisome	ECM Glycoproteins	TNNC1	Troponin C Type 1	-1.93084	1.15E-68	3.75E-66
Core matrisome	ECM Glycoproteins	CTHRC1	Collagen Triple Helix Repeat Containing 1	1.733148	1.19E-58	1.10E-56
Core matrisome	ECM Glycoproteins	ABI3BP	ABI Family, Member 3 (NESH) Binding Protein	-1.55835	3.87E-47	1.19E-45
Core matrisome	ECM Glycoproteins	PCOLCE2	Procollagen C-endopeptidase enhancer 2	-1.50257	8.48E-43	1.80E-41
Core matrisome	Proteoglycans	OGN	Osteoglycin	-1.66062	7.98E-40	1.29E-38
Matrisome-associated	ECM Regulators	MMP12	Matrix Metallopeptidase 12	2.574903	1.18E-45	3.22E-44
Matrisome-associated	ECM Regulators	MMP1	Matrix Metallopeptidase 1	2.236508	4.42E-31	3.63E-30
Matrisome-associated	ECM Regulators	ADAMTS8	ADAM Metallopeptidase With Thrombospondin Type 1 Motif, 8	-1.65606	3.61E-64	6.28E-62
Matrisome-associated	ECM-affiliated Proteins	SFTPC	Surfactant Protein C	-2.49568	1.37E-34	1.44E-33
Matrisome-associated	ECM-affiliated Proteins	GREM1	Gremlin 1, DAN Family BMP Antagonist	2.204187	3.00E-43	6.65E-42
Matrisome-associated	ECM-affiliated Proteins	SFTPA2	Surfactant Protein A2	-2.05134	7.03E-24	3.40E-23
Matrisome-associated	ECM-affiliated Proteins	SFTPD	Surfactant Protein D	-1.95274	7.74E-32	6.73E-31
Matrisome-associated	ECM-affiliated Proteins	FCN3	Ficolin (Collagen/Fibrinogen Domain Containing) 3	-1.92069	7.14E-64	1.11E-61
Matrisome-associated	Secreted Factors	WIF1	WNT Inhibitory Factor 1	-2.50673	7.70E-40	1.25E-38
Matrisome-associated	Secreted Factors	CHRDL1	Chordin-Like 1	-1.73847	8.99E-45	2.27E-43
Matrisome-associated	Secreted Factors	S100A2	S100 Calcium Binding Protein A2	1.698578	5.57E-24	2.71E-23
Matrisome-associated	Secreted Factors	CXCL2	Chemokine (C-X-C Motif) Ligand 2	-1.56452	1.85E-36	2.25E-35
Matrisome-associated	Secreted Factors	CXCL13	Chemokine (C-X-C Motif) Ligand 13	1.791842	2.13E-28	1.42E-27
Matrisome-associated	Secreted Factors	IL6	Interleukin 6	-1.60064	5.39E-37	6.84E-36
Matrisome-associated	Secreted Factors	HHIP	Hedgehog Interacting Protein	-1.72196	4.86E-50	1.94E-48
Matrisome-associated	Secreted Factors	S100A12	S100 Calcium Binding Protein A12	-1.50295	1.52E-40	2.64E-39
GO: Positive regulation of extracellular matrix constituent secretion		CPB2	Carboxypeptidase B2	-2.22141	9.71E-46	2.67E-44
GO: extracellular matrix		MAMDC2	MAM Domain Containing 2	-2.01308	4.22E-49	1.51E-47
GO: extracellular matrix		LPL	Lipoprotein Lipase	-1.55511	1.21E-44	3.04E-43
KEGG Pathway: ECM-receptor interaction		CD36	CD36 Molecule (Thrombospondin Receptor)	-1.74783	2.10E-56	1.54E-54

Supplementary Table 2. The 29-gene EPPI signature

	**Coefficient**
ABI3BP	-0.21974
ADAMTS8	-0.26293
CD36	-0.11736
CHRDL1	-0.22765
COL10A1	0.04877
COL11A1	0.17054
COL6A6	-0.12759
CPB2	-0.19902
CTHRC1	0.24657
CXCL13	0.02854
CXCL2	-0.12715
FCN3	-0.11796
GREM1	0.14724
HHIP	-0.10455
IL6	-0.08435
LPL	-0.16277
MAMDC2	-0.2063
MMP1	0.07096
MMP12	0.1102
OGN	-0.18096
PCOLCE2	-0.08783
S100A12	0.06275
S100A2	0.09983
SFTPA2	-0.06167
SFTPC	-0.07547
SFTPD	-0.11602
SPP1	0.18793
TNNC1	-0.13064
WIF1	-0.10011

Supplementary Table 3. Cox regression coefficient used in EPPI risk score computation

Lung-related diseases	References
**NSCLC**	Navab R, et al. (2011) Prognostic gene-expression signature of carcinoma-associated fibroblasts in non-small cell lung cancer. *Proc Natl Acad Sci USA* **108**:7160-7165.
**IPF**	Meltzer E, et al. (2011) Bayesian probit regression model for the diagnosis of pulmonary fibrosis: proof-of-principle. *BMC Med Genet* **4**:1.
**IPF**	DePianto D, et al. (2014) Heterogeneous gene expression signatures correspond to distinct lung pathologies and biomarkers of disease severity in idiopathic pulmonary fibrosis. *Thorax* **70**:48-56.
**IPF**	Yang I, et al. (2013) Expression of cilium-associated genes defines novel molecular subtypes of idiopathic pulmonary fibrosis. *Thorax* **68**:1114-1121.
**IPF**	Konishi K, et al. (2009) Gene Expression Profiles of Acute Exacerbations of Idiopathic Pulmonary Fibrosis. Am J Respir Crit Care Med 180:167-175.
**COPD**	Bhattacharya S, et al. (2009) Molecular Biomarkers for Quantitative and Discrete COPD Phenotypes. *Am J Respir Cell Mol Biol* **40**:359-367.
**CF**	Clarke L, Sousa L, Barreto C, Amaral M (2013) Changes in transcriptome of native nasal epithelium expressing F508del-CFTR and intersecting data from comparable studies. *Respir Res* **14**:38.

Supplementary Table 4. References of gene expression studies on other lung-related diseases

Ref	Stage	DNA/RNA/ Protein	Gene Panel	Selected based on prior survival data	HR	Lower	Upper	P-value	Validation sample size	Predictive performance	Reference
**#1**	I-II	DNA copy number	6<br>5	Yes<br>Yes	2.1<br>1.56	1.12<br>1.1	3.93<br>2.24	0.008<br>0.019	73<br>97	No<br>No	Aramburu A, Zudaire I, Pajares MJ, Agorreta J, Orta A, Lozano MD, Gúrpide A, Gómez-Román J, Martinez-Climent JA, Jassem J, Skrzypski M, Suraokar M, Behrens C, Wistuba II, Pio R, Rubio A, Montuenga LM. Combined clinical and genomic signatures for the prognosis of early stage non-small cell lung cancer based on gene copy number alterations. BMC Genomics. 2015 Oct 6;16:752.
**#2**	I, III	Gene expression	13	Yes	1.6	1.2	2.2	P < 0.001	442	No	Baty F, Facompré M, Kaiser S, Schumacher M, Pless M, Bubendorf L, Savic S, Marrer E, Budach W, Buess M, Kehren J, Tamm M, Brutsche MH. Gene profiling of clinical routine biopsies and prediction of survival in non-small cell lung cancer. Am J Respir Crit Care Med. 2010 Jan 15;181(2):181-8.
**#3**	I-III	Gene expression	50	Yes	No HR given			0.003	84	No	Beer DG, Kardia SL, Huang CC, Giordano TJ, Levin AM, Misek DE, Lin L, Chen G, Gharib TG, Thomas DG, Lizyness ML, Kuick R, Hayasaka S, Taylor JM, Iannettoni MD, Orringer MB, Hanash S. Gene-expression profiles predict survival of patients with lung adenocarcinoma. Nat Med. 2002 Aug;8(8):816-24.
**#4**	I	Gene expression	10	Yes	3.94	1.17	14.4	0.008	45	No	Bianchi F, Nuciforo P, Vecchi M, Bernard L, Tizzoni L, Marchetti A, Buttitta F, Felicioni L, Nicassio F, Di Fiore PP. Survival prediction of stage I lung adenocarcinomas by expression of 10 genes. J Clin Invest. 2007 Nov;117(11):3436-44.
**#5**	I-III	Gene expression	6	Yes	1.8<br>2.9<br>3.3<br>2.2	No 95% CI given		0.078<br>0.023<br>0.001<br>0.16	230<br>118<br>162<br>96	No<br>No<br>No<br>No	Boutros PC, Lau SK, Pintilie M, Liu N, Shepherd FA, Der SD, Tsao MS, Penn LZ, Jurisica I. Prognostic gene signatures for non-small-cell lung cancer. Proc Natl Acad Sci U S A. 2009 Feb 24;106(8):2824-8.
**#6**	I-II	Gene expression	31 (Same as #47, #48)	Proliferation genes	2.01	1.64	2.45	2.8 × 10−11	523	No	Bueno R, Hughes E, Wagner S, Gutin AS, Lanchbury JS, Zheng Y, Archer MA, Gustafson C, Jones JT, Rushton K, Saam J, Kim E, Barberis M, Wistuba I, Wenstrup J, Wallace WA, Hartman AR, Harrison DJ. Validation of a molecular and pathological model for five-year mortality risk in patients with early stage lung adenocarcinoma. J Thorac Oncol. 2015 Jan;10(1):67-73.
**#7**	All stage	Gene expression	51	Common hypoxia genes	12.84<br>2.75	1.71<br>1.15	96.5<br>6.56	0.014<br>0.023	86<br>130	No	Buffa FM, Harris AL, West CM, Miller CJ. Large meta-analysis of multiple cancers reveals a common, compact and highly prognostic hypoxia metagene. Br J Cancer. 2010 Jan 19;102(2):428-35.
**#8**	I-III	Gene expression	94	Malignant genes (proliferation, cell cycle, etc)	2.1<br>2.17<br>2.57	1.26<br>1.22<br>1.17	3.51<br>3.86<br>5.64		190<br>117<br>62	No<br>No<br>Yes	Chen DT, Hsu YL, Fulp WJ, Coppola D, Haura EB, Yeatman TJ, Cress WD. Prognostic and predictive value of a malignancy-risk gene signature in early-stage non-small cell lung cancer. J Natl Cancer Inst. 2011 Dec 21;103(24):1859-70.
**#9**	All stage	Gene expression	31	Cell cycle proliferation genes	1.71<br>1.53<br>1.82<br>2.68<br>1.88	No 95% CI given		0.0147<br>0.014<br>0.002<br>0.0008<br>0.0002	82<br>104<br>62<br>90<br>117	No	Dancik GM, Theodorescu D. Robust prognostic gene expression signatures in bladder cancer and lung adenocarcinoma depend on cell cycle related genes. PLoS One. 2014 Jan 22;9(1):e85249.
**#10**	I-II	DNA methylation	1	RASSF1A methylation	2.01	1.26	3.2		202	No	De Fraipont F, Levallet G, Creveuil C, Bergot E, Beau-Faller M, Mounawar M, Richard N, Antoine M, Rouquette I, Favrot MC, Debieuvre D, Braun D, Westeel V, Quoix E, Brambilla E, Hainaut P, Moro-Sibilot D, Morin F, Milleron B, Zalcman G; Intergroupe Francophone de Cancérologie Thoracique.. An apoptosis methylation prognostic signature for early lung cancer in the IFCT-0002 trial. Clin Cancer Res. 2012 May 15;18(10):2976-86.
**#11**	I-II	Gene expression	15 (Same as #18, #56)	Yes	2.17<br>1.61<br>1.76<br>4.19	1.12<br>0.69<br>0.97<br>0.91	4.2<br>3.77<br>3.19<br>19.15	0.018<br>0.27<br>0.058<br>0.045	127<br>54<br>128<br>43	No	Der SD, Sykes J, Pintilie M, Zhu CQ, Strumpf D, Liu N, Jurisica I, Shepherd FA, Tsao MS. Validation of a histology-independent prognostic gene signature for early-stage, non-small-cell lung cancer including stage IA patients. J Thorac Oncol. 2014 Jan;9(1):59-64.
**#12**	No prognostic performance was done.										Du L, Yamamoto S, Burnette BL, Huang D, Gao K, Jamshidi N, Kuo MD. Transcriptome profiling reveals novel gene expression signatures and regulating transcription factors of TGFβ-induced epithelial-to-mesenchymal transition. Cancer Med. 2016 Aug;5(8):1962-72.
**#13**	I-II	Gene expression	13	Yes	No HR given			0.05<br>0.00522	63<br>56	No<br>No	Guo NL, Wan YW, Bose S, Denvir J, Kashon ML, Andrew ME. A novel network model identified a 13-gene lung cancer prognostic signature. Int J Comput Biol Drug Des. 2011;4(1):19-39
**#14**	All stage	Gene expression	35	Yes	No HR given			0.04<br>0.05	24<br>111	No<br>No	Guo NL, Wan YW, Tosun K, Lin H, Msiska Z, Flynn DC, Remick SC, Vallyathan V, Dowlati A, Shi X, Castranova V, Beer DG, Qian Y. Confirmation of gene expression-based prediction of survival in non-small cell lung cancer. Clin Cancer Res. 2008 Dec 15;14(24):8213-20.
**#15**	II and III	Gene expression	6	Yes	No HR given			0.03<br>0.04<br>0.03	2 datasets (total=186)<br>45<br>58	No	Guo NL, Wan YW. Pathway-based identification of a smoking associated 6-gene signature predictive of lung cancer risk and survival. Artif Intell Med. 2012 Jun;55(2):97-105.
**#16**	All stage	Gene expression	17	Yes	2.44	No 95% CI given		0.0019	89	No	Hou J, Aerts J, den Hamer B, van Ijcken W, den Bakker M, Riegman P, van der Leest C, van der Spek P, Foekens JA, Hoogsteden HC, Grosveld F, Philipsen S. Gene expression-based classification of non-small cell lung carcinomas and survival prediction. PLoS One. 2010 Apr 22;5(4):e10312.
**#17**	All stage	Gene expression	4	Yes (invasion-associated genes)	No HR given			0.002<br>0.017	257<br>186	No<br>No	Hsu YC, Yuan S, Chen HY, Yu SL, Liu CH, Hsu PY, Wu G, Lin CH, Chang GC, Li KC, Yang PC. A four-gene signature from NCI-60 cell line for survival prediction in non-small cell lung cancer. Clin Cancer Res. 2009 Dec 1;15(23):7309-15.
**#18**	Same gene panel as #1 and #56, no further validation was done in this study. Only analytical performance was done to test assay reproducibility.										Huang S, Reitze NJ, Ewing AL, McCreary S, Uihlein AH, Brower SL, Wang D, Wang T, Gabrin MJ, Keating KE, Mulligan J, Wilson C, Davison T, McKenzie S, Tsao MS, Shepherd FA, Plamadeala V. Analytical Performance of a 15-Gene Prognostic Assay for Early-Stage Non-Small-Cell Lung Carcinoma Using RNA-Stabilized Tissue. J Mol Diagn. 2015 Jul;17(4):438-45.
**#19**	No prognostic performance was done.										Ikehara M, Oshita F, Sekiyama A, Hamanaka N, Saito H, Yamada K, Noda K, Kameda Y, Miyagi Y. Genome-wide cDNA microarray screening to correlate gene expression profile with survival in patients with advanced lung cancer. Oncol Rep. 2004 May;11(5):1041-4.
**#20**	I	Gene expression	5	Yes	3.4	1.4	9.1	0.01	62	No	Kadara H, Behrens C, Yuan P, Solis L, Liu D, Gu X, Minna JD, Lee JJ, Kim E, Hong WK, Wistuba II, Lotan R. A five-gene and corresponding protein signature forstage-I lung adenocarcinoma prognosis. Clin Cancer Res. 2011 Mar 15;17(6):1490-501.
**#21**	All stage	Gene expression	15	Yes	No HR given			0.00616	Discovery cohorts are same as validation cohorts (TCGA datasets, n=27)	No	Khirade MF, Lal G, Bapat SA. Derivation of a fifteen gene prognostic panel for six cancers. Sci Rep. 2015 Aug 14;5:13248.
**#22**	Review article of " A practical molecular assay to predict survival in resected non-squamous, non-small-cell lung cancer: development and international validation studies"/Same gene panel as #49										Kratz JR, Mann MJ, Jablons DM. International trial of adjuvant therapy in high risk stage I non-squamous cell carcinoma identified by a 14-gene prognostic signature. Transl Lung Cancer Res. 2013 Jun;2(3):222-5.
**#23**	I-II	Gene expression	7	Yes	3.2<br>5.4<br>2.5<br>2.8<br>3.8<br>1.5<br>3.5<br>3	1.3<br>1.5<br>1.5<br>1.3<br>1.5<br>0.5<br>1.8<br>1.3	7.5<br>19.5<br>4.3<br>6.1<br>9.4<br>4.2<br>6.8<br>7.4	0.0054<br>0.004<br>0.00042<br>0.0071<br>0.002<br>0.49<br>7.8E-05<br>0.0096	51<br>32<br>207<br>84<br>204<br>35<br>127<br>95	No	Krzystanek M, Moldvay J, Szüts D, Szallasi Z, Eklund AC. A robust prognostic gene expression signature for early stage lung adenocarcinoma. Biomark Res. 2016 Feb 19;4:4
**#24**	I-III	Gene expression	111	Yes	3.8	1.6	8.7	0.0008	58	No	Larsen JE, Pavey SJ, Passmore LH, Bowman R, Clarke BE, Hayward NK, Fong KM. Expression profiling defines a recurrence signature in lung squamous cell carcinoma. Carcinogenesis. 2007 Mar;28(3):760-6.

**#25**	I-III	Gene expression	54	Yes	2.2<br>3.3	1.1<br>1.4	4.7<br>7.9	0.039<br>0.044	55<br>40	No<br>No	Larsen JE, Pavey SJ, Passmore LH, Bowman RV, Hayward NK, Fong KM. Gene expression signature predicts recurrence in lung adenocarcinoma. Clin Cancer Res. 2007 May 15;13(10):2946-54.
**#26**	All stage	Gene expression	16	Apoptosis-related genes	No HR given	1.167<br>0.994<br>1.007<br>1.075	1.487<br>1.209<br>1.484<br>1.327	8.4E-06<br>0.0664<br>0.0424<br>0.00097	90<br>58<br>86<br>63	No	Lu TP, Chuang EY, Chen JJ. Identification of reproducible gene expression signatures in lung adenocarcinoma. BMC Bioinformatics. 2013 Dec 26;14:371.
**#27**	IB	Gene expression	51	Yes	No HR given			4.4E-06<br>7.3E-09<br>7.4E-05<br>9.5E-08	54<br>46<br>36<br>62	No	Lu Y, Wang L, Liu P, Yang P, You M. Gene-expression signature predicts postoperative recurrence in stage I non-small cell lung cancer patients. PLoS One. 2012;7(1):e30880.
**#28**	All stage	Gene expression	7	MUC-1 associated proliferation signatures	No HR given			0.005	84	No	MacDermed DM, Khodarev NN, Pitroda SP, Edwards DC, Pelizzari CA, Huang L, Kufe DW, Weichselbaum RR. MUC1-associated proliferation signature predicts outcomes in lung adenocarcinoma patients. BMC Med Genomics. 2010 May 6;3:16.
**#29**	All stage<br>All stage	Gene expression<br>Gene expression	12	Genomic instability signatures	2.63<br>1.55	1.61<br>1.23	4.29<br>1.96	0.05<br>0.0104	84<br>442	No<br>No	Mettu RK, Wan YW, Habermann JK, Ried T, Guo NL. A 12-gene genomic instability signature predicts clinical outcomes in multiple cancer types. Int J Biol Markers. 2010 Oct-Dec;25(4):219-28.
**#30**	All stage	Gene expression	11	Fibroblast genes	1.57<br>1.96<br>1.65	1.06<br>1.06<br>1.03	2.35<br>3.62<br>2.65	0.026<br>0.031<br>0.039	218<br>89<br>138	No	Navab R, Strumpf D, Bandarchi B, Zhu CQ, Pintilie M, Ramnarine VR, Ibrahimov E, Radulovich N, Leung L, Barczyk M, Panchal D, To C, Yun JJ, Der S, Shepherd FA, Jurisica I, Tsao MS. Prognostic gene-expression signature of carcinoma-associated fibroblasts in non-small cell lung cancer. Proc Natl Acad Sci U S A. 2011 Apr 26;108(17):7160-5.
**#31**	All stage	Gene expression	193	Yes	No HR given			0.00094<br>0.035<br>0.02<br>0.00081	256<br>125<br>58<br>117	No	Park YY, Park ES, Kim SB, Kim SC, Sohn BH, Chu IS, Jeong W, Mills GB, Byers LA, Lee JS. Development and validation of a prognostic gene-expression signature for lung adenocarcinoma. PLoS One. 2012;7(9):e44225.
	III			Based on 2-gene expression					49	Yes	
**#32**	I<br>II<br>II-III	Gene expression	5	Metabolism-related genes	No HR given			0.091<br>p<0.001<br>0.693<br>0.711	162<br>79<br>42<br>38	Yes	Pio R, Agorreta J, Montuenga LM. Prognostic signature of early lung adenocarcinoma based on the expression of ribonucleic acid metabolism-related genes. J Thorac Cardiovasc Surg. 2015 Oct;150(4):986-92.e1-11.
**#33**	All stage	Gene expression	1	IREG (An inflammation-related gene)	1.66	1	2.81	0.052	221	No	Pitroda SP, Zhou T, Sweis RF, Filippo M, Labay E, Beckett MA, Mauceri HJ, Liang H, Darga TE, Perakis S, Khan SA, Sutton HG, Zhang W, Khodarev NN, Garcia JG, Weichselbaum RR. Tumor endothelial inflammation predicts clinical outcome in diverse human cancers. PLoS One. 2012;7(10):e46104.
**#34**	I	Gene expression	67	Fibroblast specific BMP2 induced genes	2.632<br>1.389	No 95% CI given		0.0003<br>0.025	63<br>125	No	Rajski M, Saaf A, Buess M. BMP2 response pattern in human lung fibroblasts predicts outcome in lung adenocarcinomas. BMC Med Genomics. 2015 Apr 29;8:16.
**#35**	I<br>All stage<br>I<br>All stage	Gene expression	50<br>100	Yes	2.78<br>2.66<br>3.86<br>3.54	0.81<br>1.01<br>1.61<br>1.74	9.58<br>7.05<br>9.24<br>7.19	0.09<br>0.04<br>0.0012<br>0.0002	25<br>36<br>52<br>72	No	Raponi M, Zhang Y, Yu J, Chen G, Lee G, Taylor JM, Macdonald J, Thomas D, Moskaluk C, Wang Y, Beer DG. Gene expression signatures for predicting prognosis of squamous cell and adenocarcinomas of the lung. Cancer Res. 2006 Aug 1;66(15):7466-72.
**#36**	I-III	Gene expression	3	Angiogenic genes	No validation with independent data					No	Sanmartín E, Sirera R, Usó M, Blasco A, Gallach S, Figueroa S, Martínez N, Hernando C, Honguero A, Martorell M, Guijarro R, Rosell R, Jantus-Lewintre E, Camps C. A gene signature combining the tissue expression of three angiogenic factors is a prognostic marker in early-stage non-small cell lung cancer. Ann Surg Oncol. 2014 Feb;21(2):612-20.
**#37**	I	Gene expression	11	Cytokine genes	No HR given			0.08	50	No	Seike M, Yanaihara N, Bowman ED, Zanetti KA, Budhu A, Kumamoto K, Mechanic LE, Matsumoto S, Yokota J, Shibata T, Sugimura H, Gemma A, Kudoh S, Wang XW, Harris CC. Use of a cytokine gene expression signature in lung adenocarcinoma and the surrounding tissue as a prognostic classifier. J Natl [illegible]
**#38**	All stage	Gene expression	8	Yes	1.77	1.43	2.2	1.7E-07	3 combined validation sets (total=423)	Yes	Shahid M, Choi TG, Nguyen MN, Matondo A, Jo YH, Yoo JY, Nguyen NN, Yun HR, J, Akter S, Kang I, Ha J, Maeng CH, Kim SY, Lee JS, Kim J, Kim SS. An 8-gene signature for prediction of prognosis and chemoresponse in non-small cell lung cancer. Oncotarget. 2016 Dec 27;7(52):86561-86572.
**#39**	All stage	DNA copy number	46	EGFR mutation–related loci	No HR given			0.01	55	No	Shibata T, Uryu S, Kokubu A, Hosoda F, Ohki M, Sakiyama T, Matsuno Y, Tsuchiya R, Kanai Y, Kondo T, Imoto I, Inazawa J, Hirohashi S. Genetic classification of lung adenocarcinoma based on array-based comparative genomic hybridization analysis: its association with clinicopathologic features. Clin Cancer Res. 2005 Sep 1;11(17):6177-85.<br>40. Shukla S, Evans JR, Malik R, Feng FY, Dhanasekaran SM, Cao X, Chen G, Beer DG, Jiang H, Chinnaiyan AM. Development of a RNA-Seq Based Prognostic Signature in Lung Adenocarcinoma. J Natl Cancer Inst. 2016 Oct 5;109(1).
**#40**	I	Gene expression	4	Yes	2.78	1.91	11.13	<0.001	139	No	Shukla S, Evans JR, Malik R, Feng FY, Dhanasekaran SM, Cao X, Chen G, Beer DG, Jiang H, Chinnaiyan AM. Development of a RNA-Seq Based Prognostic Signature in Lung Adenocarcinoma. J Natl Cancer Inst. 2016 Oct 5;109(1).
**#41**	I-II	Gene expression	3	Yes	No HR given			0.05	26	No	Skrzypski M, Jassem E, Taron M, Sanchez JJ, Mendez P, Rzyman W, Gulida G, Raz D, Jablons D, Provencio M, Massuti B, Chaib I, Perez-Roca L, Jassem J, Rosell R. Three-gene expression signature predicts survival in early-stage squamous cell carcinoma of the lung. Clin Cancer Res. 2008 Aug 1;14(15):4794-9.
**#42**	All stage	Gene expression	10	Yes (proliferation-related genes)	1.35	1.1	1.66	0.0345	5 combined validation sets (total=862)	No	Starmans MH, Lieuwes NG, Span PN, Haider S, Dubois L, Nguyen F, van Laarhoven HW, Sweep FC, Wouters BG, Boutros PC, Lambin P. Independent and functional validation of a multi-tumour-type proliferation signature. Br J Cancer. 2012 Jul 24;107(3):508-15.
**#43**	I	Gene expression	50	Yes	No HR given			<0.01	2 combined validation cohorts (total=91)	No	Sun Z, Wigle DA, Yang P. Non-overlapping and non-cell-type-specific gene expression signatures predict lung cancer survival. J Clin Oncol. 2008 Feb 20;26(6):877-83.
**#44**	I-III	Gene expression	12	Yes	2.46	1.8	3.37	1.7E-08	5 combined validation cohorts (total=421)	Yes	Tang H, Xiao G, Behrens C, Schiller J, Allen J, Chow CW, Suraokar M, Corvalan A, Mao J, White MA, Wistuba II, Minna JD, Xie Y. A 12-gene set predicts survival benefits from adjuvant chemotherapy in non-small cell lung cancer patients. Clin Cancer Res. 2013 Mar 15;19(6):1577-86.
**#45**	All stage	Gene expression	28	Yes	No HR given			0.07	2 combined validation cohorts (total=186)	No	Wan YW, Qian Y, Rathnagiriswaran S, Castranova V, Guo NL. A breast cancer prognostic signature predicts clinical outcomes in multiple tumor types. Oncol Rep. 2010 Aug;24(2):489-94.
**#46**		Gene expression	2	CDK2 and PAK4 expression						No	Wang X, Lu Y, Feng W, Chen Q, Guo H, Sun X, Bao Y. A two kinase-gene signature model using CDK2 and PAK4 expression predicts poor outcome in non-small cell lung cancers. Neoplasma. 2016;63(2):322-9. 26774155.
**#47**	I-II	Gene expression	31 (Same as #6, #48)	Proliferation genes	2.1	1.39	3.17	0.00037	2 combined validation cohorts (total=381)	No	Wistuba II, Behrens C, Lombardi F, Wagner S, Fujimoto J, Raso MG, Spaggiari L, Galetta D, Riley R, Hughes E, Reid J, Sangale Z, Swisher SG, Kalhor N, Moran CA, Gutin A, Lanchbury JS, Barberis M, Kim ES. Validation of a proliferation-based expression signature as prognostic marker in early stage lung adenocarcinoma. Clin Cancer Res. 2013 Nov 15;19(22):6261-71.
**#48**	A cost-effectiveness analysis study using previously claimed prognostic gene panel - Same as ref #6, #47 (31 Proliferation genes)										Wong KM, Ding K, Li S, Bradbury P, Tsao MS, Der SD, Shepherd FA, Chung C, Ng R, Seymour L, Leighl NB. A Cost-Effectiveness Analysis of Using the JBR.10-Based 15-Gene Expression Signature to Guide Adjuvant Chemotherapy in Early Stage Non-Small-Cell Lung Cancer. Clin Lung Cancer. 2017 Jan;18(1):e41-e47.
**#49**	I-II	Gene expression	14 (Same as #22)	Cancer-related genes	No survival analysis was done. Risk stratification in terms of recurrence rates was compared to NCCN criteria in 52 patients.					No	Woodard GA, Gubens MA, Jahan TM, Jones KD, Kukreja J, Theodore PR, Cardozo S, Jew G, Clary-Macy C, Jablons DM, Mann MJ. Prognostic molecular assay might improve identification of patients at risk for recurrence in early-stage non-small-cell lung cancer. Clin Lung Cancer. 2014 Nov;15(6):426-32.
**#50**	I-II	Gene expression	25	Yes	No HR given			0.00601<br>0.0274	2 validation cohorts	No	Wu C, Zhang D. Identification of early-stage lung adenocarcinoma prognostic signatures based on statistical modeling. Cancer Biomark. 2017;18(2):117-123.

**#51**	All stage	Protein expression	4	Chromosomal passenger complex (CPC) proteins	2.89	1.51	5.5	0.0013	104	No	Xia R, Chen S, Chen Y, Zhang W, Zhu R, Deng A. A chromosomal passenger complex protein signature model predicts poor prognosis for non-small-cell lung cancer. Onco Targets Ther. 2015 Apr 7;8:721-6.
**#52**	All stage	Gene expression	59	Yes	1.81	No 95% CI given		0.016	111	No	Xie Y, Xiao G, Coombes KR, Behrens C, Solis LM, Raso G, Girard L, Erickson HS, Roth J, Heymach JV, Moran C, Danenberg K, Minna JD, Wistuba II. Robust gene expression signature from formalin-fixed paraffin-embedded samples predicts prognosis of non-small-cell lung cancer patients. Clin Cancer Res. 2011 Sep 1;17(17):5705-14.
					2.1			0.02	117		
**#53**	I	Gene expression	32	Yes	No HR given			0.012	299	No	Xu W, Banerji S, Davie JR, Kassie F, Yee D, Kratzke R. Yin Yang gene expression ratio signature for lung cancer prognosis. PLoS One. 2013 Jul 17;8(7):e68742.
	II-III							0.016	141	No	
**#54**	All stage	Gene expression	2	Gene Ontology signatures	No HR given			0.019	86	No	Yang X, Li H, Regan K, Li J, Huang Y, Lussier YA. Towards mechanism classifiers: expression-anchored Gene Ontology signature predicts clinical outcome in lung adenocarcinoma patients. AMIA Annu Symp Proc. 2012;2012:1040-9.
								0.001	62		
**#55**	I-II	Gene expression	39	NAMPT-influenced genes	2.88	1.69	4.95	0.00012	226	No	Zhou T, Wang T, Garcia JG. Expression of nicotinamide phosphoribosyltransferase-influenced genes predicts recurrence-free survival in lung and breast cancers. Sci Rep. 2014 Aug 22;4:6107.
					2.08	1.17	3.7	0.013	96		
**#56**	IB-II	Gene expression	15 (Same gene panel as #11, #58)	Yes	2.36	1.11	5.03	0.026	96	No	Zhu CQ, Ding K, Strumpf D, Weir BA, Meyerson M, Pennell N, Thomas RK, Naoki K, Ladd-Acosta C, Liu N, Pintilie M, Der S, Seymour L, Jurisica I, Shepherd FA, Tsao MS. Prognostic and predictive gene signature for adjuvant chemotherapy in resected non-small-cell lung cancer. J Clin Oncol. 2010 Oct 10;28(29):4417-24.
					2.01	0.92	4.41	0.08	48		
					3.18	1.4	7.25	0.006	79		
					2.02	1.06	3.86	0.033	133		
					1.96	0.95	4.02	0.062	62	Yes	
**#57**		Gene expression	12	Yes	1.87	0.65	5.43	0.247	33	No	Zhu CQ, Strumpf D, Li CY, Li Q, Liu N, Der S, Shepherd FA, Tsao MS, Jurisica I. Prognostic gene expression signature for squamous cell carcinoma of lung. Clin Cancer Res. 2010 Oct 15;16(20):5038-47.
	I				2.52	0.93	6.78	0.068	49		
					3.39	0.66	13.31	0.145	37		
					7.69	0.87	67.67	0.066	11		
	II-III				6.2	1.84	20.86	0.003	27		
					5.33	0.88	32.19	0.069	25		
**#58 (#1)**	**I-II**	**Gene expression**	**72**	**Yes (immune response-, antigen binding-, protein modification and degradation-related genes)**	**4.83**	**2.47**	**9.44**	**<0.001**	**69**	**No**	Roepman P, Jassem J, Smit EF, Muley T, Niklinski J, van de Velde T, Witteveen AT, Rzyman W, Floore A, Burgers S, Giaccone G, Meister M, Dienemann H, Skrzypski M, Kozlowski M, Mooi WJ, van Zandwijk N. An immune response enriched 72-gene prognostic profile for early-stage non-small-cell lung cancer. Clin Cancer Res. 2009 Jan 1;15(1):284-90.
**#59 (#2)**	**Same as Ref #30**										Navab R, Strumpf D, Bandarchi B, Zhu CQ, Pintilie M, Ramnarine VR, Ibrahimov E, Radulovich N, Leung L, Barczyk M, Panchal D, To C, Yun JJ, Der S, Shepherd FA, Jurisica I, Tsao MS. Prognostic gene-expression signature of carcinoma-associated fibroblasts in non-small cell lung cancer. Proc Natl Acad Sci U S A. 2011 Apr 26;108(17):7160-5.
**#59 (#3)**	**No prognostic performance/survival analysis was done in clinical samples. Only changes in cell morphology to mesenchymal phenotype were associated with mRNA and protein expression using cancer cell lines.**										Du L, Yamamoto S, Burnette BL, Huang D, Gao K, Jamshidi N, Kuo MD. Transcriptome profiling reveals novel gene expression signatures and regulating transcription factors of TGFβ-induced epithelial-to-mesenchymal transition. Cancer Med. 2016 Aug;5(8):1962-72.
**#60 (#4)**	**All stage (I-IV)**	**In contrast to the ovarian carcinomas, there is no statistically significant clinical associations between the subset of DTF–fibroblast-positive lung tumors and survival either for individual datasets or the aggregate set of lung cancers**									Chen JL, Espinosa I, Lin AY, Liao OY, van de Rijn M, West RB. Stromal responses among common carcinomas correlated with clinicopathologic features. Clin Cancer Res. 2013 Sep 15;19(18):5127-35.
**EPPI**	**I-II**	**Gene expression**	**29**	**ECM genes**	**2.1**	**1.2**	**3.6**	**0.00489**	**111**	**Yes**	
					**1.6**	**1**	**2.6**	**0.0343**	**165**		
					**3**	**1.8**	**5**	**1.7E-05**	**109**		
					**1.7**	**1.2**	**2.3**	**0.00369**	**320**		
					**1.9**	**1.1**	**3.3**	**0.0262**	**107**		
					**3.2**	**1.7**	**5.9**	**0.0001**	**109**		
					**2.3**	**1.3**	**3.8**	**0.00203**	**177**		
					**2.8**	**1.5**	**5.5**	**0.00103**	**104**		
					**1.8**	**1.2**	**3**	**0.00949**	**380**		

Supplementary Table 5. Previously reported prognostic gene signature for NSCLC

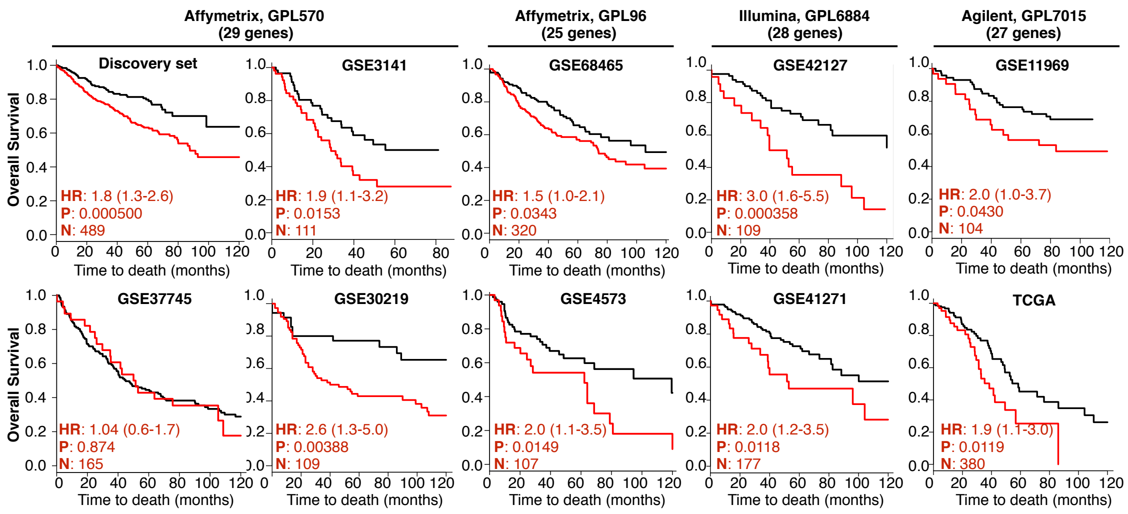

Supplementary Figure 1. Patient stratification based on EPPI gene expression using unsupervised hierarchical clustering. Patients divided into two groups had consistently different clinical outcomes of OS across multiple validation cohorts, demonstrating significant association between survival and the EPPI gene signature.

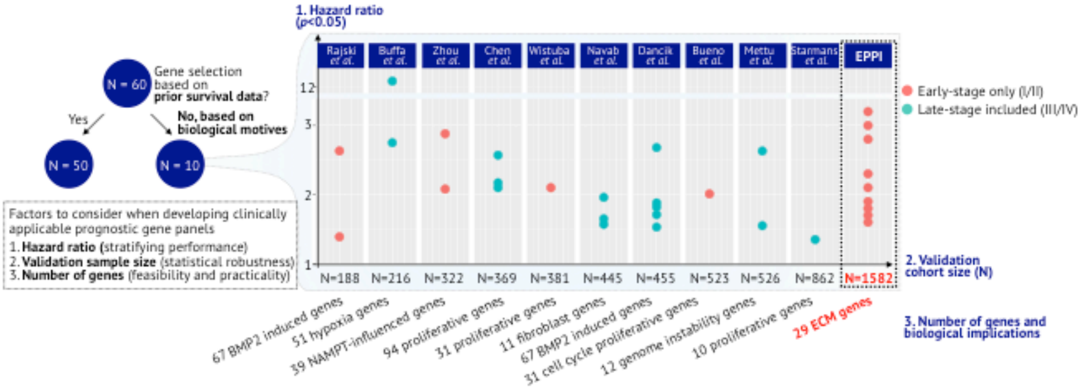

Supplementary Figure 2. Comparison to previously reported gene panels with biological implications. Of existing prognostic gene panels for NSCLC tumors, those selected based on biological motives (without prior known survival information) were selected and illustrated for HR, validation sample size, number of genes and respective biological properties, which are critical factors to be considered for development of clinically applicable multi-gene assay for prognosis.

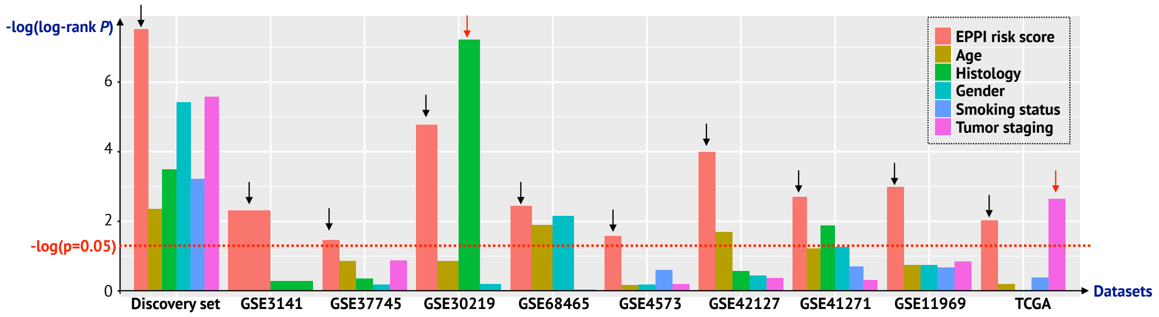

	Discovery set	GSE3141	GSE37745	GSE30219	GSE68465	GSE4573	GSE42127	GSE41271	GSE11969	TCGA
**EPPI Risk Score**										
Low-risk	193	57	136	68	161	75	82	130	62	201
High-risk	296	54	29	41	159	32	27	47	42	179
**Age**										
< 60	123	-	50	44	90	19	27	57	32	99
>= 60	284		115	65	230	88	82	119	72	281
**Histology**										
ADC	396	58	89	85	320		83	128	104	380
LCC	25		21	3				1		
SCC	67	53	55	21		107	26	48		
**Gender**										
Female	227	-	74	20	153	38	56	86	32	205
Male	262		91	89	167	69	53	91	72	175
**Smoking**										
Ever	247	-	-	-	203	99	-	158	37	318
Never	139				33	3		19	67	52
**Staging**										
IA	295	-	40	-	-	27	40	47	36	131
IB			90			46	46	84	42	130
IIA	112		6			6	6	10	5	50
IIB			29			28	17	36	21	69

Supplementary Figure 3. Comparison to previously reported gene panels with biological implications. Of existing prognostic gene panels for NSCLC tumors, those selected based on biological motives (without prior known survival information) were selected and illustrated for HR, validation sample size, number of genes and respective biological properties, which are critical factors to be considered for development of clinically applicable multi-gene assay for prognosis.
